# Acupuncture Treatment for Symptom Management in Atopic Dermatitis: A Study Protocol for a Randomized, Participant- and Assessor-Blind, Sham-Controlled Trial

**DOI:** 10.1155/2019/1907578

**Published:** 2019-05-02

**Authors:** Jung Gun Park, Hi-Joon Park, Younbyoung Chae, Yu-Kang Kim, Hyangsook Lee, Kyuseok Kim

**Affiliations:** ^1^Department of Ophthalmology, Otorhinolaryngology and Dermatology of Korean Medicine, Graduate School of Korean Medicine, Kyung Hee University, Seoul 02447, Republic of Korea; ^2^Department of Korean Medical Science, Graduate School, Kyung Hee University, Seoul 02447, Republic of Korea; ^3^Acupuncture & Meridian Science Research Centre, College of Korean Medicine, Kyung Hee University, Seoul 02447, Republic of Korea; ^4^Department of Ophthalmology, Otorhinolaryngology and Dermatology of Korean Medicine, College of Korean Medicine, Kyung Hee University, Seoul 02447, Republic of Korea

## Abstract

Atopic dermatitis (AD) is a chronic inflammatory skin disease with persistent itching, which impairs quality of life (QoL). Although various conventional treatments for AD exist, patients with AD often seek complementary and alternative therapies when conventional therapy has failed to relieve their AD symptoms or has had adverse effects. Acupuncture treatment may relieve AD symptoms, but controlled trials are needed to confirm this. Following our pilot study, which found that acupuncture treatment improves AD symptoms in mild-to-moderate AD patients, we will assess the effect of acupuncture treatment for symptom relief of AD using a trial with a complemented protocol. This is a two-arm, randomized, participant- and assessor-blinded, sham-controlled trial. A total of 36 mild-to-moderate AD patients will be randomly assigned in a 1:1 ratio to receive eight sessions twice weekly of either verum acupuncture (VA) or nonpenetrating sham acupuncture (SA) over four weeks. The primary outcome measured will be the change in the total Scoring Atopic Dermatitis (SCORAD) score. Secondary outcomes will be (1) changes in AD symptoms, QoL, dyspepsia symptoms, and electroencephalography (EEG) between baseline and week 4 and (2) changes in AD symptoms and QoL at baseline and at weeks 2, 4, and 8. This study will assess acupuncture treatment for the alleviation of AD symptoms in patients with mild-to-moderate AD. This clinical trial gas been registered in Korean Clinical Trial Registry (registration number: KCT0002796; date of registration: April 13, 2018).

## 1. Introduction

Atopic dermatitis (AD) is a chronic inflammatory skin disease characterized by intense itching, skin dryness, erythema, exudate, crust, and lichenification [1]. Globally, AD is a common health problem, and its incidence rate has increased 2-3 times in recent years in industrialized countries. Approximately, 20% of children and 3% of adults are suffering from AD [2, 3].

Initial treatments for AD are emollients, topical corticosteroids, and topical calcineurin inhibitors [4, 5]. Depending on severity, antihistamines, systemic corticosteroid, immunosuppressant, antibiotics, allergen-specific immunotherapy, or phototherapy may be used [6–8]. Complementary and alternative medicine treatments are used in patients with AD, especially when there is no response to conventional therapies or side effects occurring [9–11].

AD has been effectively treated with acupuncture, which has been shown to lack side effects when used with conventional therapies [12–16]. In animal models, acupuncture treatment resulted in decreased skin overgrowth and pruritus and improved biomarkers [5, 17]. To our knowledge, randomized controlled studies of the effectiveness of acupuncture-only treatment for AD are lacking [18], with the exception of our pilot study [19].

In this pilot study [19], a verum acupuncture (VA) treatment group showed significant alleviation of AD symptoms relative to the sham acupuncture (SA) group, including pruritus. Also, we found a positive relationship between the severity of AD symptoms and gastrointestinal (GI) symptoms such as epigastric tenderness and dyspepsia. Several studies show that GI and AD symptoms are related to unbalance of autonomic nerve system with sympathetic hyperactivity and parasympathetic dysfunction [20, 21]. Acupuncture treatment is known to affect autonomic nerve system [22]. Also, the relationship between AD and the central nervous system (CNS) has been well studied. Stress aggravates and sustains the itch-scratch cycle of AD by affecting the CNS [23]. Acupuncture treatment of AD patients may reduce brain activity of the insula, putamen, premotor and prefrontal cortical areas, which is induced by itching [24]. These findings can support the hypothesis of a “gut-brain-skin axis” suggesting a relationship between the GI tract, brain, and skin [25].

Taken together, this recent evidence suggests the need for a randomized controlled trial assessing the efficacy of acupuncture in reducing the symptoms of AD. This study protocol describes the first such study. We designed a two-arm, randomized, participant- and assessor-blind, sham-controlled trial based on our pilot study and the above recent studies. The trial aims to assess the effect of twice-weekly acupuncture treatment on the symptoms of mild-to-moderate AD in adults, including changes in quality of life (QoL), GI symptoms, and EEG.

## 2. Methods

### 2.1. Objectives

The main study objective is to determine whether or not twice-weekly acupuncture treatment improves symptoms and quality of life (QoL) in adults with mild-to-moderate AD.

Other objectives are (1) to investigate the relationship between severity of AD symptoms and GI symptoms and (2) to determine the effect of acupuncture on the CNS of AD patients using EEG.

### 2.2. Trial Design and Setting

**Figure 1:**
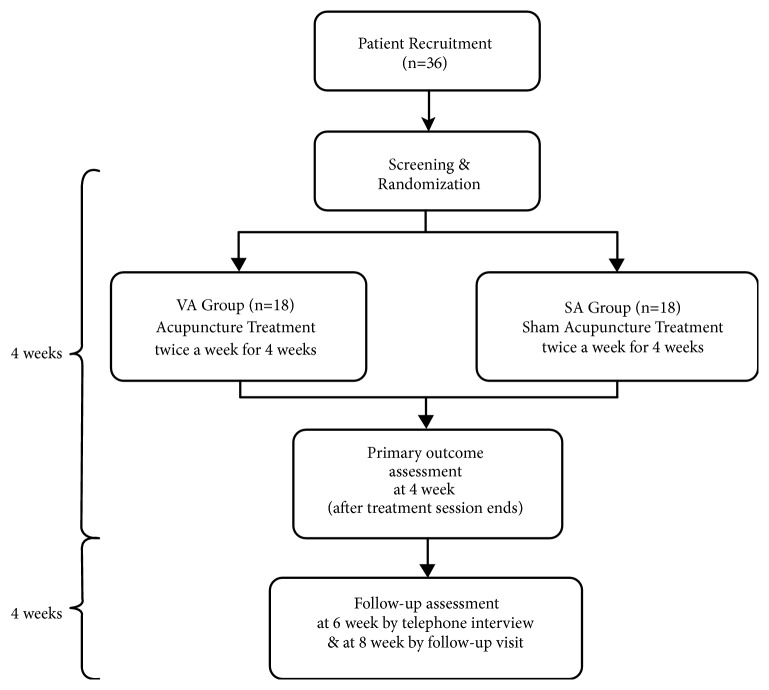
Flow of participants through the proposed trial. VA: verum acupuncture; SA: sham acupuncture.

This is a randomized, participant- and outcome assessor-blind, sham-controlled trial with a parallel group design. It will be conducted at the Kyung Hee University Korean Medicine Hospital. The flow chart of the study is presented in Figure 1.

### 2.3. Eligibility Criteria

Inclusion/exclusion criteria are as follows.

#### 2.3.1. Inclusion Criteria

(A) 19 to 65-year-old men and women able to read and write the Korean language

(B) AD diagnosed according to the Hanifin and Rajka criteria [26]

(C) Scores from 30 to 80 points on a 100 mm Visual Analog Scale (VAS) for pruritus (0, no symptom at all; 10, worst symptom ever) [27]

(D) Scores from 10 to 40 points on the objective Scoring Atopic Dermatitis (SCORAD) scale [28]

(E) Abstinence from prescribed medications for AD for the preceding month

(F) Agreement with a study protocol and willingness to sign written informed consent

(G) No participation in other clinical studies for the preceding month

#### 2.3.2. Exclusion Criteria

(A) Severely fluctuating AD symptoms

(B) Treatments (e.g., oral steroids) that may affect the symptoms of AD or that the study KMD deems inappropriate

(C) Secondary infection of AD lesions

(D) Severe mental problems

(E) Pregnancy, breastfeeding, or plans to have a baby during the study period

(F) Medical history that the study KMD considers to be inadequate

### 2.4. Recruitment of Participants

Based on our pilot study [19], we aim to recruit 36 mild-to-moderate AD patients using posters on noticeboards at the Kyung Hee Medical Center, nearby schools, and local community centers.

### 2.5. Randomization and Allocation Concealment

Participants who meet the inclusion criteria and have signed the written informed consent will be randomly assigned to either the VA or the SA group in a 1:1 ratio. An independent statistician will generate a random number table using SAS 9.2 (PROC PLAN, SAS Institute Inc., Cary, NC, USA) and send it to the administrative staff at the Acupuncture and Meridian Science Research Center (AMSRC) at Kyung Hee University. The administrative staff, who are also independent of our research team, will put each random number in a sealed, sequentially numbered, opaque envelope. Once a participant passes screening, the investigator will call the AMSRC staff, who will immediately send the investigator a document containing that participant's unique random number and group allocation, which will be kept in the trial master file.

### 2.6. Blinding and Code Breaking

Participants and outcome assessors will be kept ignorant of treatment group allocations. It is not possible to blind the acupuncturist, so the KMD who applies the acupuncture treatment will not participate in assessment of trial outcomes or in collecting and analyzing the data. During acupuncture treatment, all participants in both groups will be shielded by a screen so that they cannot see the procedures. All participants in both groups will be treated with a Park sham device to ensure ignorance of whether the acupuncture needles penetrate the skin or not. If severe side effects occur or immediate acupuncture cessation is required, the blinding code will be broken.

### 2.7. Interventions

#### 2.7.1. Acupuncture Treatment

Acupuncture treatment details are presented in Table 1 and are based on the revised Standards for Reporting Interventions in Clinical Trials of Acupuncture (STRICTA) 2010 checklist. Manual acupuncture (MA) and intradermal acupuncture (IA) will be performed for all participants in the VA group. All acupuncture treatments will be administered by one KMD with more than two years of clinical experience in Korean Medicine dermatology. This KMD will have undergone more than 10 hours of training and simulation to ensure that he is able to provide identical acupuncture treatment in accordance with the predefined protocol.

#### 2.7.2. Verum Acupuncture (VA) Group

Participants in the VA group will receive eight acupuncture treatments twice a week for a total of four weeks. All VA group patients will receive acupuncture needling at six fixed acupoints. Up to 10 additional acupoints will be used, depending on each participant's daily general conditions. The six fixed acupoints are LI11, ST36, and PC6 bilaterally. Additional acupoints include ST43, GB41, LI2, TE3, SI3, TE6, SI2, BL66, LR3, SP3, ST43, and GB41 for gastric stuffiness or dyspepsia; LI2 and GB41 for tenderness around ST25, diarrhea, or constipation; TE3 and SI3 for fullness in the chest and hypochondrium; TE3 and TE6 for lower abdominal pain and tenderness on CV17; SI3 and GB41 for lower abdominal pain and dry skin; SI2 and BL66 for lower abdominal pain and heat in the upper body and cold in the lower body; and LR3 and SP3 for pain in the hypogastric region with darkness of the sublingual collateral vessels. Disposable sterile stainless needles (0.25 x 40 mm; Dongbang Acupuncture Inc., Bundang, Seongnam, Korea) will be used. Acupuncture needles will be inserted to depths of 5 to 30 mm, depending on the acupoint, and will remain in place for 15 minutes.

After removal of acupuncture needles, three acupoints will be treated with IA using 1.5 mm press tack needles (Haeng Lim Seo Won Medical Co., Korea). These are bilateral LI11 and auricular-Shenmen contralaterally to the side with more severe symptoms. Participants will be instructed by the study KMD to press LI11 for more than three minutes when they experience severe itching.

#### 2.7.3. Sham Acupuncture (SA) Group

Participants in the SA group will receive sham acupuncture treatment eight times twice a week for four weeks. Unlike the VA group, SA group patients will receive sham acupuncture treatment at only six fixed control points. These bilateral acupoints are as follows: 1 to 2 cm proximal and 1 cm medial to LI7, 1 cm proximal and 1 cm medial to LI11, and 1 cm proximal and 1 cm lateral to ST36. To maintain participant blinding, a nonpenetrating sham device (Park sham acupuncture needles, AcuPrime Co., Ltd., Exeter, UK) will be used and left in place for 15 minutes.

After removal of sham acupuncture needles, the IA procedure used in the VA group will be replaced with application of needle-free, stainless steel rings that do not penetrate the skin. These will be attached to three control points: 1 cm proximal and 1 cm medial to LI11 bilaterally and finger point in the ear contra-laterally to the side with more severe symptoms. Unlike the VA group, SA group patients will not be instructed to press the control point.

#### 2.7.4. After Treatment

Two weeks after the eighth visit for the final acupuncture treatment, a telephone interview will be conducted to assess the severity of AD and dyspepsia symptoms, side effects after acupuncture treatment, and whether or not other treatments were used during the trial. During a final visit, four weeks after the eighth visit, the participant will complete a final evaluation. No other treatments will be allowed during the study period.

### 2.8. Compliance and Discontinuation

Frequent hospital visits may be a burden to the participant, resulting in withdrawal from the study. Based on the results of our pilot study [19], we determined that completion of six to eight treatment sessions was sufficient for a participant to be considered compliant and yield acceptable data. For dropouts who do not fulfil this criterion, the last observed data will be used for the analysis. The research coordinator will keep in contact with participants to encourage them to continue participation to the end of the trial.

### 2.9. Outcome Measures

A researcher who is independent of this study will evaluate the outcomes. The schedule is shown in Table 2. The primary measurable outcome is the change in SCORAD (total) before treatment (baseline) and after (at four weeks) Treatment. Secondary outcomes are as follows: (1) baseline to four-week changes in AD symptoms, QoL, GI symptoms, or EEG and (2) changes between baseline and second, fourth, and eighth weeks in AD symptoms and QoL. Measurable values are as follows: SCORAD Index, Eczema Area and Severity Index (EASI), Patient Oriented Eczema Measure (POEM), Dermatology Life Quality Index (DLQI), abdominal Pressure Pain Threshold (PPT) using an algometer developed in the Korea Institute of Oriental Medicine [29], questionnaires about dyspepsia symptoms (Nepean Dyspepsia Index-Korean version, Adequate Relief of functional dyspepsia pain and discomfort, and VAS for dyspepsia), and EEG.

#### 2.9.1. Scoring Atopic Dermatitis (SCORAD)

SCORAD was developed in 1993 by the European Task Force on Atopic Dermatitis as a useful scoring system that assesses the severity of AD by its extent, intensity, and subjective symptoms [28, 30, 31]. The extent component of SCORAD is assessed using the rule of nines for AD lesions. The intensity component of SCORAD is divided into erythema, edema and papules, oozing and crust, excoriation, lichenification, and dryness. Subjective symptom measures are the degrees of pruritus and sleep disturbance assessed on the VAS.

#### 2.9.2. Eczema Area and Severity Index (EASI)

EASI is a tool for evaluating the severity of AD symptoms. Like the SCORAD index, it is known as a useful evaluation tool [30, 32, 33]. Body parts are divided into four parts (head and neck, trunk, upper limb, and lower limb) to evaluate the extent of AD lesions. The severity of symptoms (erythema, edema and papules, excoriation, and lichenification) is then evaluated.

#### 2.9.3. Patient Oriented Eczema Measure (POEM)

POEM is a simple, easily understood, and well-validated tool for evaluating the severity of AD symptoms in the preceding week. It is known to be appropriate for use in outpatient clinic or clinical trial settings [30, 34].

#### 2.9.4. Dermatology Life Quality Index (DLQI)

DLQI is a well-validated clinical research tool that is widely used to assess the QoL of patients with dermatologic symptoms. It evaluates the effect of skin disease on QoL during the preceding week and consists of 10 questions [35, 36].

#### 2.9.5. Abdominal Examination Using Pain Pressure Threshold (PPT)

In Korean Medicine, KMDs use abdominal examination for diagnosis, choice of treatment methods, and/or evaluation of prognosis [37]. We will standardize and describe abdominal examination quantitatively using the PPT algometer. The PPT algometer used in our pilot study [19] was found to be unsuitable for abdominal examination, so a more appropriate algometer developed in the Korea Institute of Oriental Medicine will be used [29]. The outcome assessor will apply the algometer before and after acupuncture treatment to measure the patient's abdominal tenderness at four acupuncture points (CV17, CV12, and bilateral ST25). Pressure at each acupoint will be delivered at a constant force and velocity following the computerized guide. Each measurement will be performed three times for 1 minute [38].

#### 2.9.6. Nepean Dyspepsia Index (NDI)

NDI is a reliable scale for functional dyspepsia which evaluates dyspepsia symptoms and related QoL. NDI consists of period, severity, and degree of distress of 15 dyspepsia-related symptoms [39, 40]. In this study, participant will be asked to answer the Korean version of NDI (NDI-K) [41].

#### 2.9.7. Adequate Relief of Functional Dyspepsia Pain and Discomfort (AR)

AR assesses improvement in overall functional dyspepsia symptoms [42]. Subjects will be asked to answer the following question: “Have you experienced appropriate relief (more than 50% reduction) of the pain or discomfort associated with your dyspepsia after the last acupuncture treatment?”

#### 2.9.8. VAS for Functional Dyspepsia

Using 100 mm VAS (0 mm for no discomfort to 100 mm for the worst discomfort), we will measure a degree of overall dyspeptic symptoms.

#### 2.9.9. Credibility/Blinding Test

In order to evaluate the credibility of the study participants' acupuncture treatment, a questionnaire-type credibility test will be conducted at the first and eighth treatment visits and the follow-up visit [43]. The following question will also be asked once at the eighth treatment visit when the last treatment is given: “Which group do you think you belonged to? The treatment group or the control group, and why?” This question aims to determine the direction and degree of blinding between the VA group and the SA group [44].

#### 2.9.10. Measurement of Electroencephalography (EEG)

Subjective feelings of itchiness in AD may be induced or enhanced by watching someone scratch himself or herself. Brain signals associated with somatosensory perception may be changed by contagious itch [45]. Visual stimuli depicting scratching behavior can induce changes in oscillatory potentials measured with EEG. This study is designed to explore the relationship between neuronal oscillations and subjective itchiness induced by contagious itch.

All patients will be asked to evaluate how much itchiness they feel after watching video clips of a person either scratching or performing a control movement. Their evaluation will use a visual analog scale ranging from 0 to 10. Continuous EEG will be recorded using Brain Products EEG amplifiers with a 32-channel ActiCAP system according to the international 10-20 system. Event-related spectral perturbation (ERSP) methods will be applied to EEG data, while patients are watching the video clips. Through time-frequency analysis, the suppression of mu activity will be measured over central-parietal regions. Mu rhythm represents frequencies in the range of 8-13 Hz, and the suppression of mu activity can be observed over the primary sensorimotor cortex when an action is performed, observed, or imagined [46]. The EEG results will be compared within each patient between scratching and control movement videos. We hypothesize that the suppression of mu activity over the sensorimotor cortex will be decreased after the treatment in the VA group but not in the SA group.

### 2.10. Sample Size Calculation

Sample size calculation was carried out using G*∗*Power 3.1.9.2 for Windows 10. The primary measurable outcome in this study is the change in SCORAD (total) before treatment (at baseline) and at the end of treatment (four weeks). We derived the sample size of this study using information from our pilot study [19] showing a change in total SCORAD at baseline and after treatment of 13.0 ± 9.9 and 4.6 ± 5.0 in the VA and SA groups, respectively, and effect size d was 1.1. Based on this, the critical t-value was 2.0 for 28 degrees of freedom, and the noncentrality parameter *δ* was 3.0 (two-tailed independent* t*-test) at 5% significance level, with 80% power and 20% dropout rate. Required sample size was calculated as 18 per group.

### 2.11. Statistical Analysis

Statistical analyses will be performed by an independent statistician who is unaware of group assignments, using IBM SPSS for Windows (Version 21.0). Continuous variables will be presented as mean ± 1 standard deviation, and bivariate variables will be presented as numbers (%). The effect of the intervention will be assessed as the change in outcome measures between baseline and four weeks (after treatment sessions end) and through eight weeks (after follow-up period ends), using a repeated-measures analysis of variance (ANOVA) and *χ*2 test. The difference in effect between VA and SA will be assessed by comparing changes in the SCORAD (total) index, the AD symptoms, QoL, dyspepsia symptoms, and EEG outcomes, using the independent* t*-test or Mann–Whitney *U* test on continuous variables and *χ*2 test or Fisher's exact test on categorical variables.

ITT (intention-to-treat) analysis will be used for all participants who have undergone at least one acupuncture treatment. For missing values, the last observation carried forward (LOCF) method will be applied. PP (per protocol) analysis will be used for only the participants who have completed the entire course of the test exactly as specified in the trial plan. Primary evaluation of the intervention effect will be based on the results of the ITT analysis.

### 2.12. Participant Safety

During the informed consent procedures, study participants will be informed of potential adverse events such as local bleeding, subcutaneous hematoma, or pain associated with acupuncture treatment. Adverse events that occur after acupuncture treatment will be assessed at each visit and after treatment is concluded and will be recorded in the case report form (CRF). Appropriate actions will be taken to minimize adverse events. If a serious adverse event occurs, the blinding code will be broken and the case immediately reported to the clinical trial director and the Institutional Review Board (IRB) at Kyung Hee University Korean Medicine Hospital. If necessary, part or all of the trial will be suspended until further instructions are available. Should a participant experience adverse events from trial participation, she/he will be treated at the research institute's expense until recovery.

### 2.13. Confidentiality

After enrollment, all records that identify the research participants will be kept confidential and can only be viewed by researchers. All documents related to the study will be identified by unique codes rather than participants' names to protect confidentiality. All data and records related to the trial will be securely locked under the curation of the administration for three years after publication of the trial results and then destroyed.

### 2.14. Quality Control

All study researchers will receive centralized and unified training before the study. This study will be conducted under the periodic monitoring of AMSRC at Kyung Hee University and Korean Medicine Clinical Trial Center at Kyung Hee University Korean Medicine Hospital. This protocol follows Standard Protocol Items: Recommendations for Interventional Trials (SPIRIT) (see Additional File 1 in Supplementary Materials) [47].

If a protocol modification is required due to a serious adverse event or unanticipated problem, the representatives of Kyung Hee University Korean Medicine Hospital and the AMSRC will discuss the matter fully to take the appropriate steps.

## 3. Discussion

The incidence of AD is increasing in developed countries, and the disorder affects both children and adults [2, 3]. Conventional therapies, such as the use of topical corticosteroids, may be ineffective or lead to adverse side effects. Acupuncture treatment has been performed in AD patients, but controlled clinical trials to assess its effectiveness are lacking. In our pilot study [19], acupuncture was shown to be more effective in relieving the symptoms of AD than sham acupuncture, and the severity of AD was associated with the presence of GI symptoms.

The relationships between skin, brain, and GI system health [25] and between AD and the CNS [23, 24] suggest a role for acupuncture in relieving the symptoms of interrelated disorders of these organ systems. To assess this possible role, we designed a new confirmatory study with a complementary medicine protocol that builds on the results of our pilot study [19] and these studies.

We will recruit 36 mild-to-moderate AD patients for this study, scoring 30 to 80 points on a 100 mm VAS for pruritus (0, no symptom at all; 10, worst symptom ever) [27] and 10 to 40 points in the object SCORAD scores [28]. KM treatment such as acupuncture and herbal medicine may also relieve the symptoms of severe AD patients [12, 13]. However, in this trial, patients will receive only acupuncture treatment. To remove factors that may confound or mask the effect of acupuncture, no antipruritic treatments such as topical steroids or antihistamines will be allowed. For participants' safety and convenience, patients with severe AD symptoms will be excluded. The difficulty of assessing relief from AD in patients with slight symptoms, who score less than 30 points on a 100 mm VAS for pruritus or less than 10 points in the object SCORAD scores, leads us to exclude them too.

In this study, we will use the same acupoints as in the pilot study [19]. These acupoints were selected based on traditional meridian theory [48], clinical experience, and consensus among experts in acupuncture and AD. Case studies using acupoints similar to this study (LI11, ST36, etc.) [13, 49] have shown relief of AD symptoms. The pilot study [19] showed significant improvement in itching and SCORAD score, further supporting our decision to use the same acupoints in the trial proposed here.

The dose of acupuncture treatment was also determined based on our pilot study [19], which compared two different doses of acupuncture treatment given two and three times weekly for four weeks, with the same doses of SA treatment. While both acupuncture groups significantly alleviated AD symptoms relative to the SA group, we found no significant difference between the twice- and thrice-weekly groups. To reduce the time and financial costs associated with hospital visits, we therefore decided that twice-weekly acupuncture treatment would be sufficient to detect treatment effects between VA and SA groups.

An appropriate sham control procedure is key to the success of an acupuncture RCT study. We designed the sham control to avoid physiological effects that might be induced by percutaneous needle insertion and to avoid specific acupoints. First, to avoid needle penetration, we will use the Park sham acupuncture device (AcuPrime Co., Ltd., Exeter, UK), which is known to be an adequate sham control for acupuncture [50], for administering both sham and real needles. Second, to avoid specific acupoints, we will use different acupoints in the two groups. Third, a blinding curtain will be used to ensure that acupuncture procedures take place out of sight of the participants. Last, a blinding questionnaire will also be used at the end of all acupuncture treatment sessions to assess effectiveness of blinding.

There is a battery of outcome measures in this study. They can be divided into five categories: (1) measures of the severity of AD symptoms: SCORAD, VAS (Pruritus), VAS (Insomnia), EASI, and POEM, (2) a measure of the QoL affected by AD: DLQI, (3) measures of the severity of dyspepsia symptoms: NDI-K, AR, VAS of dyspepsia, and abdominal PPT, (4) measures of central nervous system responses of AD patients: EEG, and (5) a credibility test, blinding test, and assessment of adverse effects.

In our previous pilot study [19], SCORAD (Total), VAS (Pruritus), VAS (Insomnia), POEM, DLQI, and credibility test scores differed significantly between the VA and SA groups over time. The changes in SCORAD (Objective), SCORAD (Total), and EASI were significantly different between the VA and SA groups and between weeks 0 and 4. SCORAD (Total) was the only indicator that differed both between groups and over time. Also, SCORAD is one of the most sensitive indicators for assessing the severity of AD. Therefore, in this study, changes in SCORAD (Total) between weeks 0 and 4 are set as the primary outcome measure. SCORAD (Objective), VAS (Pruritus), VAS (Insomnia), EASI, DLQI, and POEM, which were significantly different between VA and SA groups in the pilot study, are set as the secondary outcomes.

The unique finding of our pilot study [19] was a significant effect of epigastric tenderness and dyspepsia on the main indicators of AD, especially SCORAD, VAS (Pruritus), VAS (Insomnia), POEM, and DLQI. This supports the proposed “gut-brain-skin axis” linking skin disease with CNS and digestive symptoms [25]. Also, several studies have shown that AD patients with GI symptoms have significant improvement in the AD symptoms when their GI symptoms are treated [51, 52]. For all these reasons, we added NDI-K, AR, VAS of dyspepsia, and abdominal PPT, which are quantitative indicators of indigestion, to the secondary outcomes of the trial that we propose here.

This study protocol will provide an important and thorough assessment of the effectiveness of twice-weekly acupuncture treatment in improving the symptoms of mild-to-moderate AD in adults. It also aims to explore changes in QoL affected by AD, changes in the degree of GI symptoms, and EEG as secondary outcomes.

## Figures and Tables

**Table 1:** Acupuncture treatment details based on the checklist for STRICTA 2010. STRICTA: Standards for Reporting Interventions in Clinical Trials of Acupuncture; MA: Manual Acupuncture; IA: Intradermal Acupuncture; PTN: Press Tack Needle; AD: Atopic Dermatitis; VA: Verum Acupuncture; SA: Sham Acupuncture; KMD: Korean Medicine Doctor.

Item	Detail
(1) Acupuncture Rationale	(1a) Style of acupuncture
	(i) MA and IA using PTN based on traditional meridian theory
	(1b) Reasoning for treatment provided, based on historical context, literature sources, and/or consensus methods, with references where appropriate
	(i) Partially individualized MA treatment and IA treatments based on the traditional meridian theory, clinical experience, and consensus by the experts in acupuncture and AD
	(1c) Extent to which treatment was varied
	MA: Partially individualized, that is, 6 fixed points plus optional points according to individual symptoms
	IA: Fixed treatment

(2) Details of Needling	(2a) Number of needle insertions per subject per session (mean and range where relevant) MA: 6 to 19 body acupoints per participant per session IA: 2 body acupoints and 1 auricular acupoint per participant per session (2b) Names (or location if no standard name) of points used (uni/bilateral) MA:
	(i) fixed points: PC6, LI11, ST36 bilaterally
	(ii) optional points: ST43, GB11, LI2, TE3, TE6, SI2, SI3, BL66, LR3, SP3 contralaterally (GB41, TE3, SI3 can be applied bilaterally according to the signs or symptoms of the patient)
	IA: LI11 bilaterally and auricular shenmen contralaterally
	(2c) Depth of insertion, based on a specified unit of measurement or on a particular tissue level MA: From 5 to 30 mm, perpendicular to skin surface IA: 1.5 mm, perpendicular to skin surface (2d) Response sought (e.g., de qi or muscle twitch response) MA: “de qi” sensation IA: None (2e) Needle stimulation (e.g., manual, electrical) MA: Manual stimulation, needle rotation with thumb and index fingers for the first 10-15 seconds IA: Participants will be educated to press PTNS at LI11 for more than 3 minutes when they feel severe itching (2f) Needle retention time MA: 15 minutes IA: 1-2 days until PTN falls off (2g) Needle type (diameter, length, and manufacturer or material) MA: A sterilized stainless steel needle (0.25 x 40 mm, Dongbang Acupuncture Inc., Bundang, Seongnam, Korea) IA: A hypoallergic PTN (1.5 mm, 10 x 10 mm adhesive tape, Haeng Lim Seo Won Medical Co., Korea)

(3) Treatment Regimen	(3a) Number of treatment sessions
	(i) 8 sessions
	(3b) Frequency and duration of treatment sessions
	(i) Twice a week for 4 weeks, 15 minutes for each session

(4) Other Components of Treatment	(4a) Details of other interventions administered to the acupuncture group (e.g. moxibustion, cupping, herbs, exercises, lifestyle advice)
	(i) Lifestyle advice will be given to all participants
	(ii) Any other interventions will be prohibited during study period
	(4b) Settings and context of treatment, including instructions to practitioner and information and explanations to patients
	(i) Participants will be informed about acupuncture treatment in the study as follows: “In this study, you will be randomly allocated to VA group or SA group. Acupoints will be selected based on traditional Korean medicine textbook and AD-related reports. Also, additional acupoints can be used according to individual conditions, if needed.”

(5) Practitioner Background	(5) Description of participating acupuncturists (qualification or professional affiliation, years in acupuncture practice, other relevant experience)
	(i) KMD who has clinical experience in Korean Medicine dermatology more than 2 years. The practitioner KMD will have undergone more than 10 hours of training and simulation workshop to ensure that he is able to provide identical acupuncture treatment in accordance with a predefined protocol.

(6) Control and Comparator Interventions	(6a) Rationale for the control or comparator in the context of the research question, with sources that justify this choice
	(i) A control group (SA) will be treated with sham acupuncture using Park sham acupuncture needles and nonpenetrating sham PTNs
	(6b) Precise description of the control or comparator. If sham acupuncture or any other type of acupuncture-like control is used, provide details as for items (1) to (3) above
	MA: Park sham acupuncture needles and devices will be used in same environment as in the VA group. However, fixed acupoints will be different: a point 1 to 2 cm proximal and 1 cm medial to LI7, a point 1 cm proximal and 1 cm medial to LI11, and a point 1 cm proximal and 1 cm lateral to ST36, each bilaterally
	IA: Nonpenetrating sham PTNs, which were designed and validated for blinding for our study, will be used as a control in same environment as in the VA group. The same sized stainless steel rings without needles will be attached to three control points: a point 1 cm proximal and 1 cm medial to LI11, bilaterally, and finger point in the ear contralaterally.

**Table 2:** *Study Schedule*. VAS: Visual Analog Scale; SCORAD: Scoring Atopic Dermatitis; EASI: Eczema Area and Severity Index; POEM: Patient Oriented Eczema Measure; DLQI: Dermatology Life Quality Index; PPT: Pain Pressure Threshold; NDI-K: Nepean Dyspepsia Index-Korean version; AR: Adequate Relief of functional dyspepsia pain and discomfort; EEG: Electroencephalography. ● represents treatment applied at that particular interval; 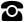: participant contacted by telephone.

Treatment and measure	Baseline	Treatment period				Follow-up period			
		1 week	2 weeks	3 weeks	4 weeks	5 weeks	6 weeks	7 weeks	8 weeks
Demographics	●								

Physical Examination	●								

Medical history	●								

VAS for itch	●	●	●	●	●		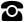		●

VAS for sleep loss	●	●	●	●	●		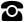		●

VAS for dyspepsia	●	●	●	●	●		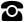		●

SCORAD assessment	●	●	●	●	●				●

EASI assessment		●	●	●	●				●

POEM assessment		●	●	●	●				●

DLQI assessment		●	●	●	●				●

PPT evaluation		●	●		●				

NDI-K assessment		●			●				●

AR assessment				●	●		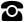		●

Credibility test		●			●				

Blinding test					●				

EEG assessment		●			●				

Acupuncture Treatment		●	●	●	●				

Adverse Events		●	●	●	●		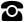		●

## Data Availability

Data and materials are available upon request from the corresponding authors.

## Abbreviations

AD: Atopic Dermatitis
AMSRC: Acupuncture and Meridian Science Research Center
AR: Adequate Relief of functional dyspepsia pain and discomfort
DLQI: Dermatology Life Quality Index
EASI: Eczema Area and Severity Index
EEG: Electroencephalogram
IA: Intradermal Acupuncture
IRB: Institutional Review Board
ITT analysis: Intention-to-treat analysis
KM: Korean Medicine
KMD: Korean Medicine Doctor
MA: Manual Acupuncture
NDI-K: Nepean Dyspepsia Index-Korean version
POEM: Patient Oriented Eczema Measure
PP analysis: Per protocol analysis
PPT: Pain Pressure Threshold
PTN: Press Tack Needle
SA group: Sham Acupuncture group
SCORAD: Scoring Atopic Dermatitis
STRICTA: Standards for Reporting Interventions in Clinical Trials of Acupuncture
QoL: Quality of Life
VA group: Verum Acupuncture group
VAS: Visual Analog Scale.

## Supplementary Materials

Supplementary Materials SPIRIT 2013 Checklist: recommended items to address in a clinical trial protocol and related documents∗.
